# Supplementary material for: Whole Transcriptome Sequencing Reveals Drought Resistance-Related Genes in Upland Cotton
Source: Genes (Basel). 2022 Jun 27;13(7):1159. doi: 10.3390/genes13071159 (PMC9318479; doi:10.3390/genes13071159)
Supplement: Supplementary file 1 [file genes-13-01159-s001.zip › genes-1755694-supplementary.pdf]

Table S1. Primer sequences of differentially expressed genes.

Gene ID	Forward Primer (5′-3′)	Reverse Primer (5′-3′)
Gohir.A11G156000	AGCGTTGAAGGCTACAGAGGAGAG	TCAGTTCACACGAAGCAGCACAC
Gohir.A07G220600	TCCTCTCCCACTTCCCGAACATAG	CAGCACGCACTCCACCACAC
Gohir.D05G218300	ATGTCCAACGCCTGTTCCCA	AGCCGCCCATGAATCGAGAA
Gohir.A11G141800	TTCACCTTCTTCCTTGCAGCG	GTGCAGGTTGGAGCTGTGGAT
Gohir.D02G166900	TTTTCAGGCACTTGCTCGGC	ACCTTCAGTTCCTCTTCGGCA
Gohir.A05G304100	AAAGGTTCCAGCAGACGCCA	AATGCAGCCTCCGCTAGGTC
Gohir.A09G158200	ATATGGTGGATTGCGGGCCA	GGCGCGTGCTGTTTTCGTAT
Gohir.A13G152200	TCCTGACCAGCAAGTGGTGT	GCAATCAGGTGCCTCAACCC
Gohir.D05G218200	TGGAGGTGTTGGACAAGCGA	TCTGCAAGGGTCAGCCTCAA
Actin	ATCCTCCGTCTTGACCTTG	TGTCCGTCAGGCAACTCAT

Table S2. Experimental grouping.

Sample Number	Organisation Site	Different Treatments	Experimental Grouping
XJ0hR1	blade	0 h treatment of drought-resistant materials	control group
XJ0hR2	blade		control group
XJ0hR3	blade		control group
XJ0hS1	blade	0 h treatment of drought-sensitive materials	control group
XJ0hS2	blade		control group
XJ0hS3	blade		control group
XJ12hR1	blade	12 h treatment of drought-resistant materials	test group
XJ12hR2	blade		test group
XJ12hR3	blade		test group
XJ12hS1	blade	12 h treatment of drought-sensitive materials	test group
XJ12hS2	blade		test group
XJ12hS3	blade		test group
XJ24hR1	blade	24 h treatment of drought-resistant materials	test group
XJ24hR2	blade		test group
XJ24hR3	blade		test group
XJ24hS1	blade	24 h treatment of drought-sensitive materials	test group
XJ24hS2	blade		test group
XJ24hS3	blade		test group

Note: A total of 18 materials were obtained; R, drought-resistant material Xinluzhong No. 82; S, drought-sensitive material Kexin No. 1; 0 h, 12 h, and 24 h represent different processing times; and R1, R2, R3, S1, S2, and S3 represent different biological repetitions.

Table S3. Gohir.A11G156000.v2.1. expression levels.

**XJ0hR1_Count**	**XJ0hR2_Count**	**XJ0hR3_Count**	**XJ12hR1_Count**	**XJ12hR2_Count**	**XJ12hR3_Count**	**XJ0hR1_FPKM**	**XJ0hR2_FPKM**	**XJ0hR3_FPKM**	**XJ12hR1_FPKM**	**XJ12hR2_FPKM**	**XJ12hR3_FPKM**	***p*-Value**	**log2FC**	**Regulated**
4003	2474	3860	9632	7280	6983	16.35	13.45	16.82	46.05	40.35	37.97	1.22×10^{-7}	0.922	up
XJ0hS1_Count	XJ0hS2_Count	XJ0hS3_Count	XJ12hS1_Count	XJ12hS2_Count	XJ12hS3_Count	XJ0hS1_FPKM	XJ0hS2_FPKM	XJ0hS3_FPKM	XJ12hS1_FPKM	XJ12hS2_FPKM	XJ12hS3_FPKM	*p*-value	log2FC	regulated
3044	2876	2983	6772	7408	7241	16.60	14.66	15.43	35.89	40.32	38.38	1.12×10^{-12}	0.784	up
XJ12hR1_Count	XJ12hR2_Count	XJ12hR3_Count	XJ24hR1_Count	XJ24hR2_Count	XJ24hR3_Count	XJ12hR1_FPKM	XJ12hR2_FPKM	XJ12hR3_FPKM	XJ24hR1_FPKM	XJ24hR2_FPKM	XJ24hR3_FPKM	*p*-value	log2FC	regulated
9632	7280	6983	2018	2688	3567	46.05	40.35	37.97	10.84	14.24	17.42	6.38×10^{-13}	−1.239	down
XJ12hS1_Count	XJ12hS2_Count	XJ12hS3_Count	XJ24hS1_Count	XJ24hS2_Count	XJ24hS3_Count	XJ12hS1_FPKM	XJ12hS2_FPKM	XJ12hS3_FPKM	XJ24hS1_FPKM	XJ24hS2_FPKM	XJ24hS3_FPKM	*p*-value	log2FC	regulated
6772	7408	7241	2537	3302	2516	35.89	40.32	38.38	13.28	17.41	12.90	1.74×10^{-12}	−1.101	down

Table S4. Gohir.A07G220600.v2.1. expression levels.

**XJ0hR1_Count**	**XJ0hR2_Count**	**XJ0hR3_Count**	**XJ12hR1_Count**	**XJ12hR2_Count**	**XJ12hR3_Count**	**XJ0hR1_FPKM**	**XJ0hR2_FPKM**	**XJ0hR3_FPKM**	**XJ12hR1_FPKM**	**XJ12hR2_FPKM**	**XJ12hR3_FPKM**	***p*-Value**	**log2FC**	**Regulated**
3279	2181	3912	164	287	156	8.600	7.616302	10.946612	0.503461	1.019193	0.542571	1.80×10^{-35}	-4.078519682	down
XJ0hS1_Count	XJ0hS2_Count	XJ0hS3_Count	XJ12hS1_Count	XJ12hS2_Count	XJ12hS3_Count	XJ0hS1_FPKM	XJ0hS2_FPKM	XJ0hS3_FPKM	XJ12hS1_FPKM	XJ12hS2_FPKM	XJ12hS3_FPKM	*p*-value	log2FC	regulated
2364	3325	3246	370	281	154	8.282	10.884507	10.782553	1.259015	0.97912	0.52093	8.70×10^{-31}	-3.832399289	down
XJ12hR1_Count	XJ12hR2_Count	XJ12hR3_Count	XJ24hR1_Count	XJ24hR2_Count	XJ24hR3_Count	XJ12hR1_FPKM	XJ12hR2_FPKM	XJ12hR3_FPKM	XJ24hR1_FPKM	XJ24hR2_FPKM	XJ24hR3_FPKM	*p*-value	log2FC	regulated
164	287	156	4256	2021	2709	0.503	1.019193	0.542571	14.684347	6.871942	8.496771	3.55×10^{-19}	4.003553295	up
XJ12hS1_Count	XJ12hS2_Count	XJ12hS3_Count	XJ24hS1_Count	XJ24hS2_Count	XJ24hS3_Count	XJ12hS1_FPKM	XJ12hS2_FPKM	XJ12hS3_FPKM	XJ24hS1_FPKM	XJ24hS2_FPKM	XJ24hS3_FPKM	*p*-value	log2FC	regulated
370	281	154	2738	3093	2151	1.259	0.97912	0.52093	9.204302	10.476203	7.083376	3.89×10^{-23}	3.435904968	up

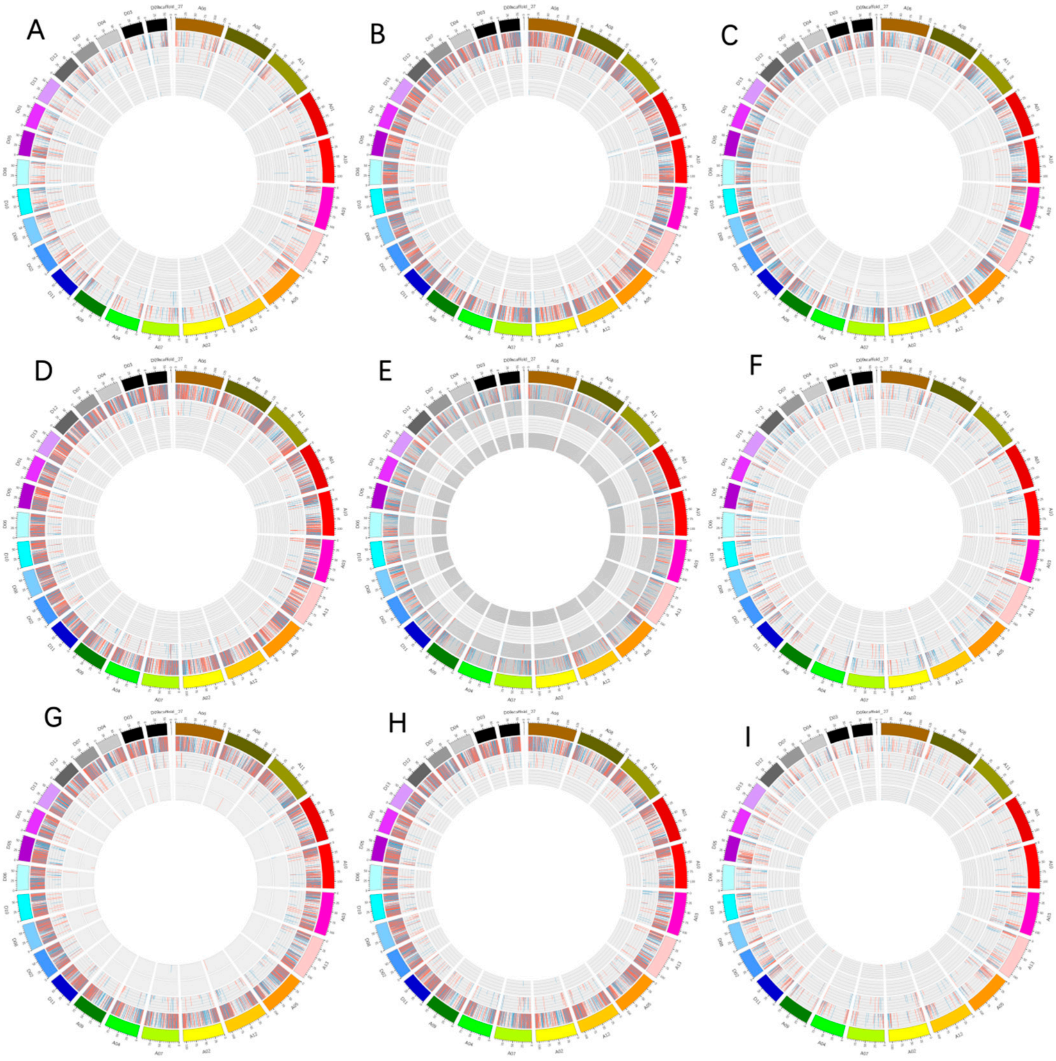

Figure S1. Circos diagrams for different levels of significance across RNA types. Note: The outermost circle shows chromosome information, followed by mRNA, lncRNA, circRNA, and miRNA. In the diagrams for each group, red represents upregulation, blue represents downregulation, and the height represents significance (−log10(FDR or *p*-value)).

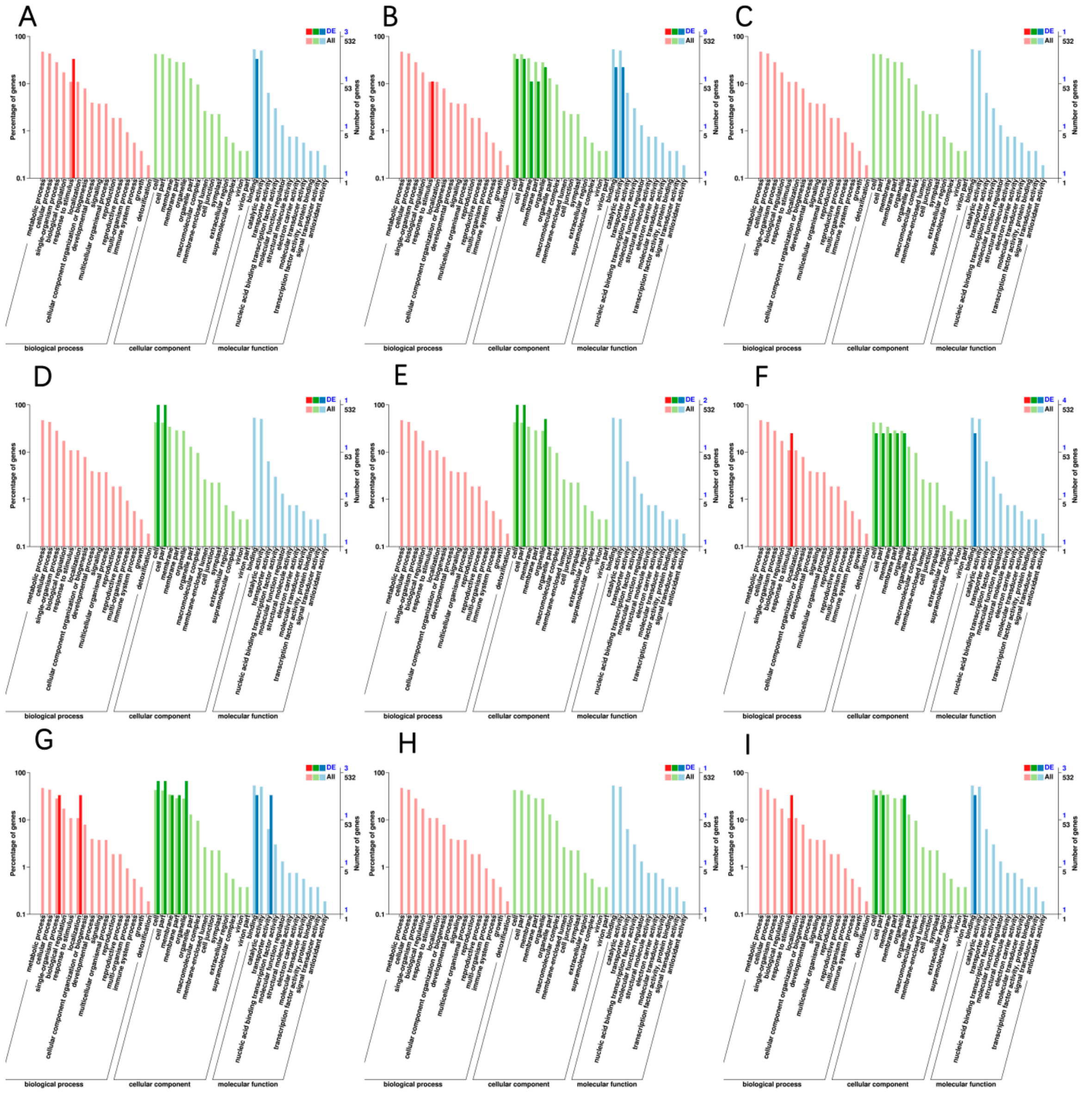

Figure S2. Annotation statistics diagrams of the GO secondary nodes of the different circRNA source genes in each comparison group. Note: The abscissa is the GO classification, the left ordinate is the percentage of the number of genes, and the right is the number of genes. This figure shows the gene annotation status of each secondary function of GO based on circRNA source genes and all genes. It reflects the status of each secondary function in the two backgrounds. The secondary functions with obvious proportional differences indicate that circRNA source genes are compared with all genes, indicating that if the enrichment trend of genes is different, this function is important. A:XJ0hR1_XJ0hR2_XJ0hR3_vs_XJ0hS1_XJ0hS2_XJ0hS3;B:XJ0hR1_XJ0hR2_XJ0hR3_vs_XJ12hR1_XJ12hR2_XJ12hR3;C:XJ0hR1_XJ0hR2_XJ0hR3_vs_XJ24hR1_XJ24hR2_XJ24hR3;D:XJ0hS1_XJ0hS2_XJ0hS3_vs_XJ12hS1_XJ12hS2_XJ12hS3;E:XJ0hS1_XJ0hS2_XJ0hS3_vs_XJ24hS1_XJ24hS2_XJ24hS3;F:XJ12hR1_XJ12hR2_XJ12hR3_vs_XJ12hS1_XJ12hS2_XJ12hS3;G:XJ12hR1_XJ12hR2_XJ12hR3_vs_XJ24hR1_XJ24hR2_XJ24hR3;H:XJ12hS1_XJ12hS2_XJ12hS3_vs_XJ24hS1_XJ24hS2_XJ24hS3;I:XJ24hR1_XJ24hR2_XJ24hR3_vs_XJ12hS1_XJ12hS2_XJ12hS3.

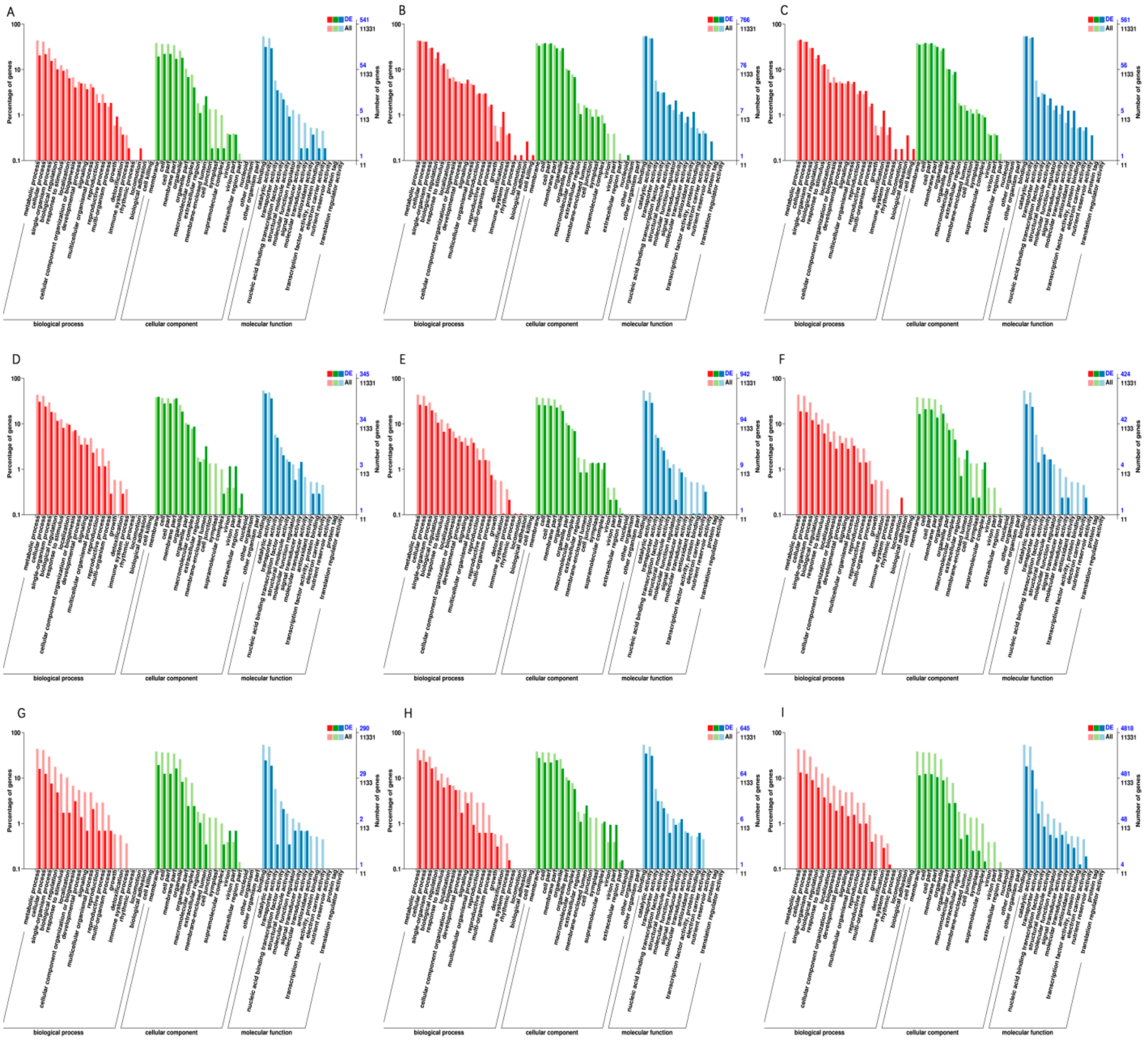

Figure S3. GO annotation classification statistics of differentially expressed miRNA target genes. Note: The abscissa is the GO classification, the left ordinate is the percentage of the number of genes, and the right is the number of genes. This figure shows the gene enrichment of each secondary function of GO based on the differentially expressed miRNA target genes and all the genes, reflecting the status of each secondary function in the two backgrounds. The secondary functions with obvious proportional differences indicate that circRNA source genes are compared with all genes, indicating that if the enrichment trend of genes is different, this function is important. (**A**) XJ0hR1_XJ0hR2_XJ0hR3_vs_XJ0hS1_XJ0hS2_XJ0hS3; (**B**) XJ0hR1_XJ0hR2_XJ0hR3_vs_XJ12hR1_XJ12hR2_XJ12hR3; (**C**) XJ0hR1_XJ0hR2_XJ0hR3_vs_XJ24hR1_XJ24hR2_XJ24hR3; (**D**) XJ0hS1_XJ0hS2_XJ0hS3_vs_XJ12hS1_XJ12hS2_XJ12hS3; (**E**) XJ0hS1_XJ0hS2_XJ0hS3_vs_XJ24hS1_XJ24hS2_XJ24hS3; (**F**) XJ12hR1_XJ12hR2_XJ12hR3_vs_XJ12hS1_XJ12hS2_XJ12hS3; (**G**) XJ12hR1_XJ12hR2_XJ12hR3_vs_XJ24hR1_XJ24hR2_XJ24hR3; (**H**) XJ12hS1_XJ12hS2_XJ12hS3_vs_XJ24hS1_XJ24hS2_XJ24hS3; (**I**) XJ24hR1_XJ24hR2_XJ24hR3_vs_XJ12hS1_XJ12hS2_XJ12hS3.

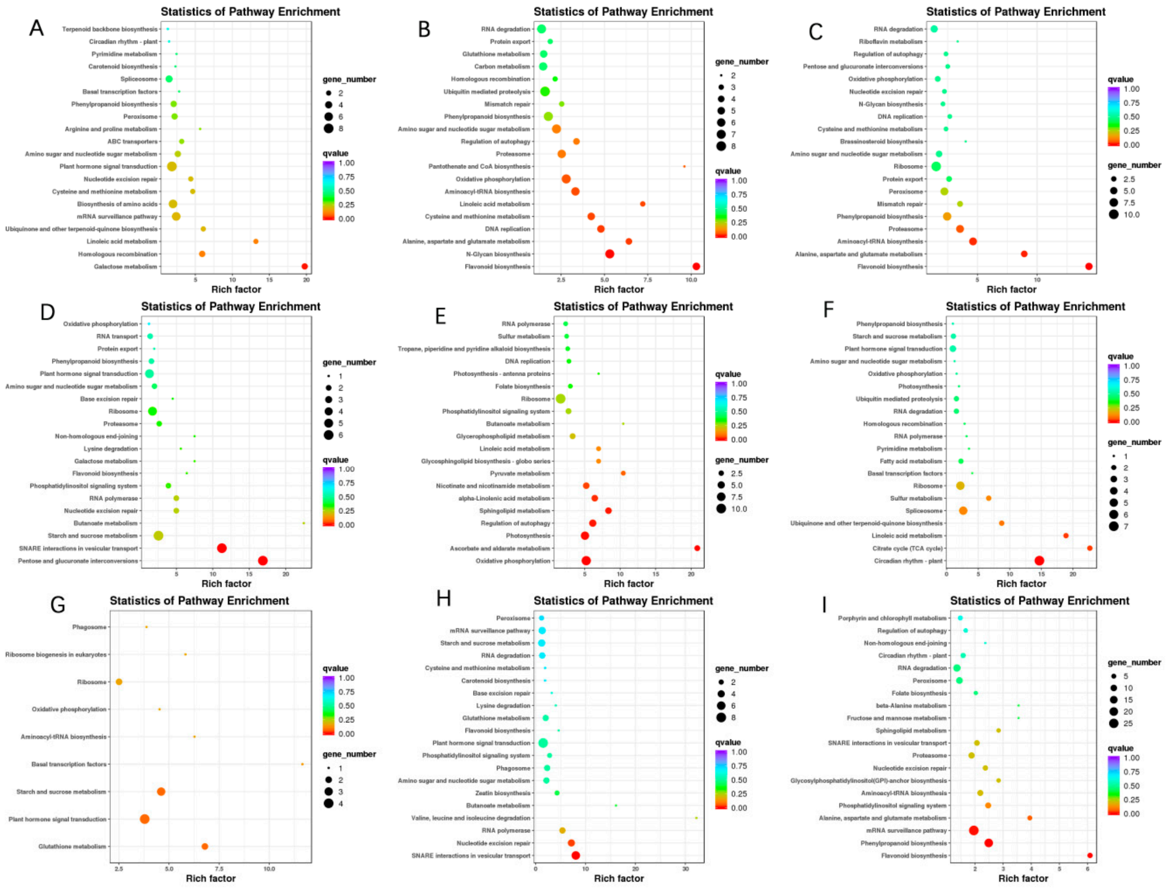

Figure S4. KEGG pathway enrichment scatter diagram of differentially expressed miRNA target genes. Note: Each graph in the figure represents a KEGG pathway; the abscissa is the enrichment factor, which represents the proportion of the number of differentially expressed miRNA target genes annotated to a pathway among the total number of genes annotated to the pathway. The larger the value is, the more significant the enrichment level of the differentially expressed miRNA target gene in the pathway. The ordinate is −log10 (Q value), where the Q value is the *p*-value after multiple hypothesis testing correction; therefore, the larger the ordinate is, the higher the enrichment significance of differentially expressed miRNA target genes in this pathway. (**A**) XJ0hR1_XJ0hR2_XJ0hR3_vs_XJ0hS1_XJ0hS2_XJ0hS3; (**B**) XJ0hR1_XJ0hR2_XJ0hR3_vs_XJ12hR1_XJ12hR2_XJ12hR3; (**C**) XJ0hR1_XJ0hR2_XJ0hR3_vs_XJ24hR1_XJ24hR2_XJ24hR3; (**D**) XJ0hS1_XJ0hS2_XJ0hS3_vs_XJ12hS1_XJ12hS2_XJ12hS3; (**E**) XJ0hS1_XJ0hS2_XJ0hS3_vs_XJ24hS1_XJ24hS2_XJ24hS3; (**F**) XJ12hR1_XJ12hR2_XJ12hR3_vs_XJ12hS1_XJ12hS2_XJ12hS3; (**G**) XJ12hR1_XJ12hR2_XJ12hR3_vs_XJ24hR1_XJ24hR2_XJ24hR3; (**H**) XJ12hS1_XJ12hS2_XJ12hS3_vs_XJ24hS1_XJ24hS2_XJ24hS3; (**I**) XJ24hR1_XJ24hR2_XJ24hR3_vs_XJ12hS1_XJ12hS2_XJ12hS3.

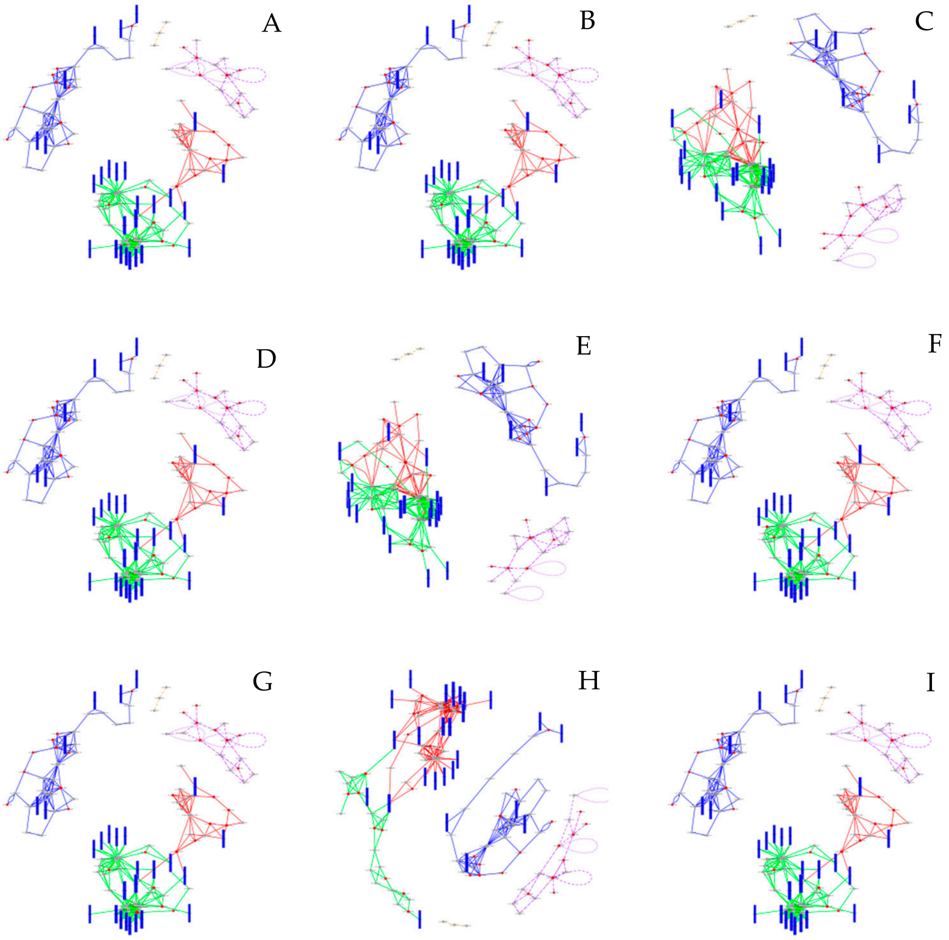

Figure S5. The top five most enriched pathways in each comparison group. Note: Each dot represents a gene, each rectangle represents a pathway, and the line represents the relationship between genes, and between genes and other pathways. The colours of different lines indicate that the relationship comes from different pathways. The red dots are the key genes. (**A**) XJ0hR1_XJ0hR2_XJ0hR3_vs_XJ0hS1_XJ0hS2_XJ0hS3; (**B**) XJ0hR1_XJ0hR2_XJ0hR3_vs_XJ12hR1_XJ12hR2_XJ12hR3; (**C**) XJ0hR1_XJ0hR2_XJ0hR3_vs_XJ24hR1_XJ24hR2_XJ24hR3; (**D**) XJ0hS1_XJ0hS2_XJ0hS3_vs_XJ12hS1_XJ12hS2_XJ12hS3; (**E**) XJ0hS1_XJ0hS2_XJ0hS3_vs_XJ24hS1_XJ24hS2_XJ24hS3; (**F**) XJ12hR1_XJ12hR2_XJ12hR3_vs_XJ12hS1_XJ12hS2_XJ12hS3; (**G**) XJ12hR1_XJ12hR2_XJ12hR3_vs_XJ24hR1_XJ24hR2_XJ24hR3; (**H**) XJ12hS1_XJ12hS2_XJ12hS3_vs_XJ24hS1_XJ24hS2_XJ24hS3; (**I**) XJ24hR1_XJ24hR2_XJ24hR3_vs_XJ12hS1_XJ12hS2_XJ12hS3.
